# Supplementary material for: Identification and functional analysis of non-coding regulatory small RNA FenSr3 in Bacillus amyloliquefaciens LPB-18
Source: PeerJ. 2023 May 15;11:e15236. doi: 10.7717/peerj.15236 (PMC10194069; doi:10.7717/peerj.15236)
Supplement: Supplemental Information 4 [file peerj-11-15236-s004.zip › KO/CK-vs-T1_map/map04972.html]

KEGG PATHWAY: Pancreatic secretion - Reference pathway


|  |  |
| --- | --- |
| **Pancreatic secretion - Reference pathway** |  |

[
Pathway menu
| Organism menu
| Pathway entry
| Show description
| User data mapping
]

The pancreas performs both exocrine and endocrine functions. The exocrine pancreas consists of two parts, the acinar and duct cells. The primary functions of pancreatic acinar cells are to synthesize and secrete digestive enzymes. Stimulation of the cell by secretagogues such as acetylcholine (ACh) and cholecystokinin (CCK) causes the generation of an intracellular Ca2+ signal. This signal, in turn, triggers the fusion of the zymogen granules with the apical plasma membrane, leading to the polarised secretion of the enzymes. The major task of pancreatic duct cells is the secretion of fluid and bicarbonate ions (HCO3-), which neutralize the acidity of gastric contents that enter the duodenum. An increase in intracellular cAMP by secretin is one of the major signals of pancreatic HCO3- secretion. Activation of the CFTR Cl- channel and the CFTR-dependent Cl-/HCO3- exchange activities is responsible for cAMP-induced HCO3- secretion.

|  |  |  |
| --- | --- | --- |
| Reference pathway | 184% 150% 122% 100% 82% 67% 55% | 图片下载 |
